# Minding the gaps: assessing and addressing clinical research core competencies across a network of Canadian cancer centres

**DOI:** 10.3389/fphar.2023.1294335

**Published:** 2023-12-08

**Authors:** Stephen Sundquist, Diana Kato, Raisa Chowdhury, Christine Samara, Janet E. Dancey

**Affiliations:** ^1^ Canadian Cancer Clinical Trials Network, Toronto, ON, Canada; ^2^ Clinical Research Program, Odette Cancer Centre, Sunnybrook Research Institute, Toronto, ON, Canada; ^3^ Faculty Associate, Clinical Research Management & Regulatory Science Programs, Edson College of Nursing and Health Innovation, Arizona State University, Tempe, AZ, United States; ^4^ Member, JTF for Clinical Trial Competency Committee, MRCT of Brigham and Women’s Hospital and Harvard, Boston, MA, United States; ^5^ Canadian Cancer Trials Group, Queen’s University, Kingston, ON, Canada

**Keywords:** core competency, workforce development, clinical research, professional development, research training

## Abstract

The Canadian Cancer Clinical Trials Network (3CTN, the Network), established in 2014 to address the decline in academic cancer clinical trials’ (ACCT) activity, has successfully achieved incremental year-over-year accrual targets as well as implemented recognized performance measures and supports for improving efficiency and quality of trial activities at member sites across Canada. As part of efforts to address ongoing challenges of staff recruitment, retention, and turnover in academic institutions that have been more recently exacerbated by the pandemic, the Network’s Performance Strategy Sub-Committee (PSC) oversaw surveys of site clinical research professionals intended to capture workforce development status and identify knowledge gaps using the Joint Task Force Core Competency Framework (JTF CCF) as the standard basis for assessment. Accountable to the 3CTN Management Committee, the PSC consists of clinical research operations experts across Canada responsible for overseeing implementation and monitoring progress of this initiative. Staff at 3CTN’s adult sites evaluated and reported trial personnel core competencies and gaps according to each domain/leveled competency statement of the framework. The most frequently noted competency gaps were in the domains of: Investigational Product Development and Regulation (28%); Scientific Concepts and Research Design (16%); and Study and Site Management (14%). Reported data was compiled and represented in the 3CTN Core Competency Report, developed as a web-based, interactive tool enabling members and stakeholders to filter data to enumerate and quantify workforce competency gaps at their site, within their node of affiliated sites, or across the national Network. Concurrently, an environmental scan and review of education resources was conducted and reviewed by the PSC. Embedded links to curated learning and development resources were incorporated into the report and associated with each domain/leveled competency statement to provide ready access to high-quality learning and development resources where needed. In the remaining years of its current strategic plan, 3CTN will continue to monitor, develop collaborative initiatives to target prioritized clinical research competency gaps and create opportunities for ongoing assessment and reporting by sites to capture changes in workforce core competencies over time.

## 1 Introduction

**FIGURE 1:**
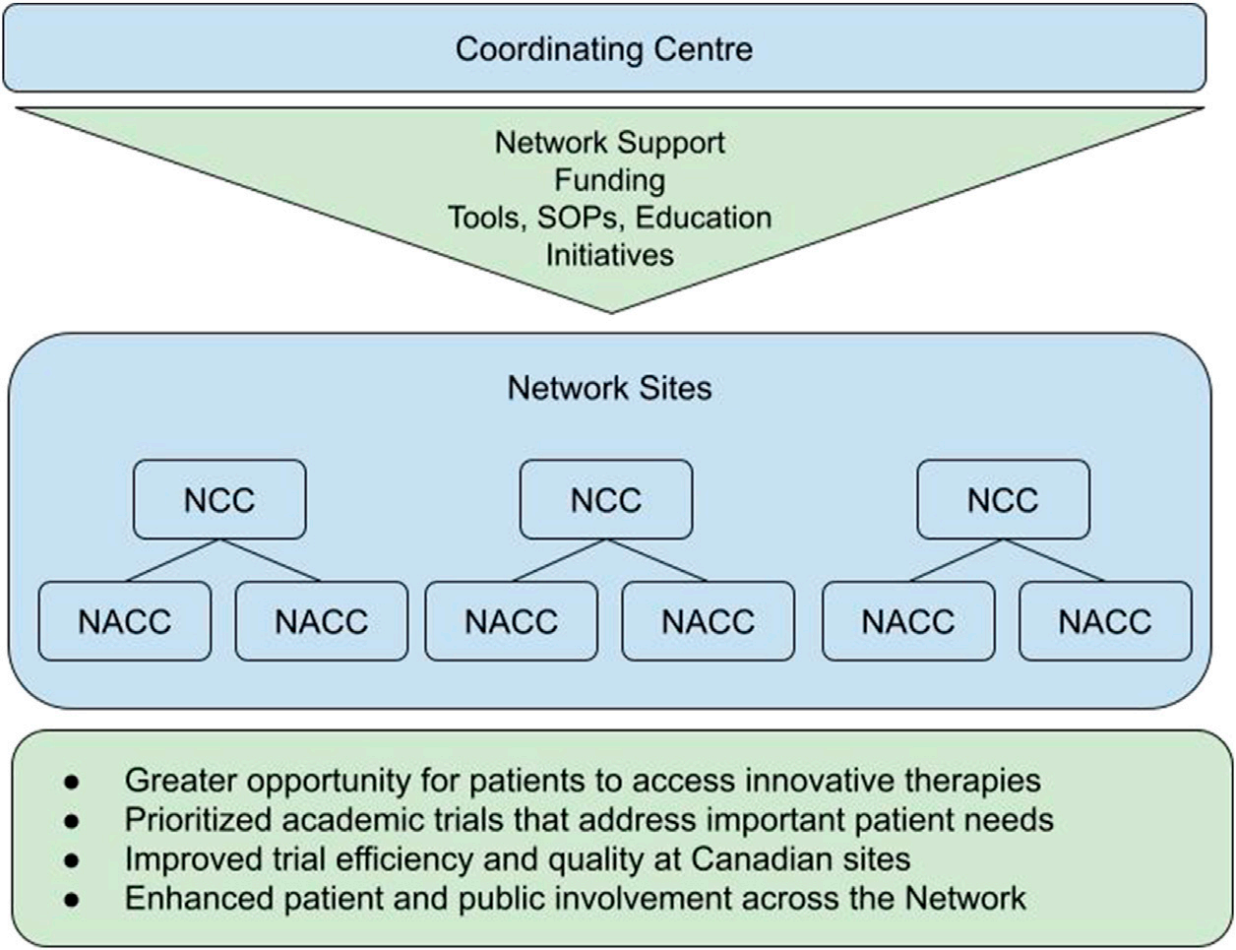
3CTN organizational framework.

The Canadian Cancer Clinical Trials Network (3CTN, the Network) was established in 2014 to address a national decline in academic cancer clinical trials activity (Canadian Cancer Research Alliance, 2011). Network objectives include providing support for clinical trial infrastructure at cancer centres and hospitals across Canada to ensure the accrual and efficient execution of ACCTs. Current 3CTN members are clinician investigators and clinical research professionals in trial units of 39 adult cancer centres and hospitals. In some provinces, larger Network Cancer Centers (NCC) also support trial unit operations at Network Affiliated Cancer Centres (NACC) within their region (Figure 1), with a total of 11 NCC and 28 NACCs. 3CTN membership at NCCs typically consists of ∼5–10 clinical research professionals within the trial unit, whereas NACC teams comprise of ∼two to five staff. The Network’s communications, operations processes and infrastructure enable member centres to work collaboratively, exchange knowledge and best practices, and develop research competencies for improved trial conduct. Collectively, Network member sites have successfully achieved incremental improvements in year-over-year accrual targets as well as implemented standard performance measures and supports for improving trial activation times and quality within member sites across Canada (Xu et al., 2021).

Since the commencement of its current 5-year strategic period in 2022, 3CTN-member Cancer Centres have consistently cited unprecedented staffing challenges affecting trial unit performance, capacity, and development progress. Healthcare staff reallocation to frontline care in the first months of the COVID-19 pandemic and prolonged periods of uncertainty and flux impacting local operating practices and the clinical trial environment contributed to widespread burnout and turnover (Knapke et al., 2022a; Knapke et al., 2022b; Sundquist et al., 2022; Freel et al., 2023). Departures due to elective retirement decisions or internal transfers to other roles offering more flexible work arrangements were among the reasons cited. Already a common occurrence within academic institutions, moves to roles within pharma, cancer agencies or other external organizations were more frequently reported at this time. More competitive compensation packages, flexible work arrangements and/or professional development opportunities offered are frequent draws.

While issues of retention and resulting challenges for sustaining research core competencies present significant challenges for all sites, most Canadian cancer centre trial units tend to be small, with trials conduct often undertaken by only a handful of coordinators and research nurses. This makes them especially vulnerable to turnover where onboarding, orientation and core training of new hires can take months to complete and draws heavily on managers’ and co-workers’ already dealing with increased workloads. Typically, turnover has longer-term impacts on sites’ operating capacity, trial performance and quality improvement goals as lost team-level efficiencies and core competencies are re-established.

**FIGURE 2:**
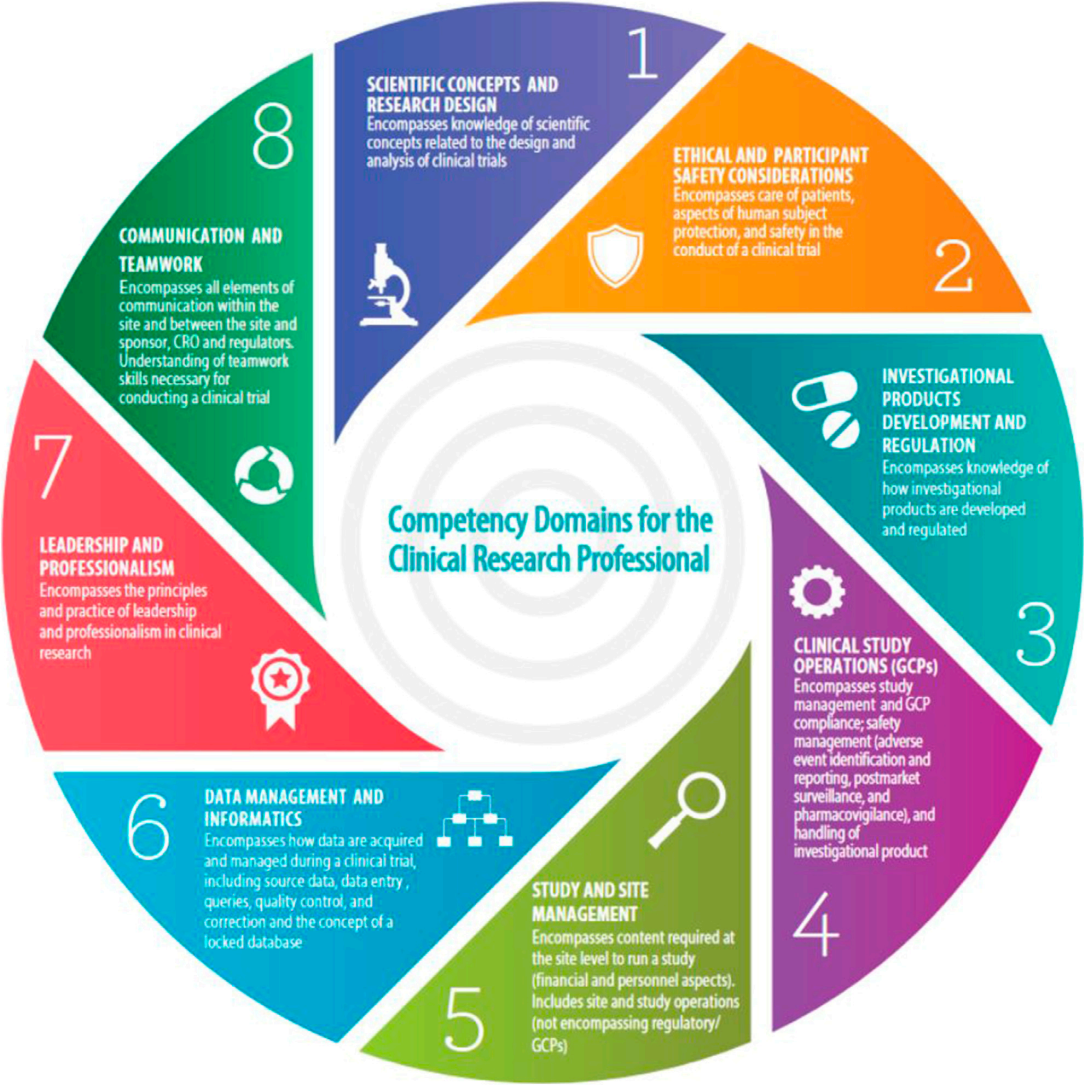
Joint task force core competency framework. Source: https://mrctcenter.org/clinical-trial-competency/.

As part of a strategic priority to support member sites’ workforce recruitment, retention, and development, and guided by its Performance Strategy Sub-Committee (PSC), the 3CTN Coordinating Centre team undertook a systematic approach to:

1. Identify Network-wide clinical research professional core competency gaps through a survey of 3CTN member sites using JTF CCF as a standard basis of assessment. The framework defines the competency domains and associated skills necessary to conduct high quality, ethical and safe clinical trials was used as a standard basis of assessment (Figure 2) (Sonstein and Jones, 2018).
2. Identify resources aligned with core competency domains to support clinical trials research education and training for staff.
3. Develop an interactive web-based report for sites to review competency results and directly access links to available education and training resources for identified gap areas.

## 2 Methods

Beginning in 2021 and as part of the membership application for the Network’s 2022–2027 business period, 3CTN centres evaluated and reported staff core competencies and gaps in detail according to each domain/leveled competency statement of the JTF core competency framework (see survey in, Supplementary Table S2). On behalf of their teams, site leads (e.g., trial unit manager, team leads drawn from various clinical research professional roles) reported on overall capacity to perform competencies associated with each leveled competency statement. An inability to perform described activities was identified as a gap. Relevant guidance documents and other reference materials were also included to illustrate competency standards and help guide assessments for each domain. Individual sites were expected to draw from their results to identify competency domains requiring improvement over the period as part of deliverables tied to their Network agreements.

Results from all sites’ assessments were inputted into 3CTN’s central database, forming the basis of the PSC’s evaluation and selection of national priorities for professional development and serving as a reference for tracking progress over time. Members of the PSC were asked to select an initial set of leveled competency statement gaps identified from the site assessments to be considered for Network-level support. The basis of selection considered the prevalence of gaps cited by respondents, the expected level of effort required to develop competencies within multiple trial site teams, and anticipated impact (high to low) that could be realized in each area. Also considered were the existence of accessible training or learning resources, and potential benefit to 3CTN priorities for improving trial capacity, performance, and accrual. All clinical research unit team members at Network sites were then requested in a follow-up survey to score priorities for each of the leveled competency statements identified by the PSC based on expected benefit from competency development to their role/team performance (see survey in, Supplementary Table S3). A comment box was included to capture additional suggestions for training topics.

**FIGURE 3:**
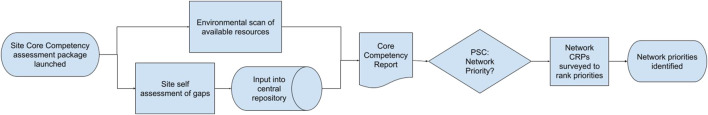
Overview of the process to identify Network-wide core competency priorities.

The PSC also oversaw an environmental scan and review of available educational materials to curate a trusted list of resources for developing competencies associated with each leveled competency statement for all domains. An initial search of clinical research training and education programs was conducted using repositories from known sources of clinical research training and education programs from CITI Program, Society of Clinical Research Associates (SoCRA), Association of Clinical Research Professionals (ACRP), Network of Networks (N2), Canadian Clinical Trials Group (CCTG). A general search using clinical research core competency keywords was also completed to capture any additional resources. Each were categorized by core competency domain, resource type (i.e., course, webinar, document) and reviewed by the PSC. A total of 89 resources were identified and incorporated via links within the 3CTN Core Competency Report, created to provide a visual representation of the pan-Canadian Network survey results, overall and by site type (NCC, NACC), within the interactive, web-based resource. Engagement of Network clinical research professionals during development helped validate the web-based tool’s utility and usability for end users. An overview of the process is provided in Figure 3.

## 3 Results

During the initial 3CTN renewal application process, trial unit leads from 41 Network sites reported gaps associated with leveled competency statements under each domain. Refer to Table 1 for a summary of the proportion of identified competency areas by domain as reported from the site assessment and Supplementary Table S1 for a further breakdown by leveled competency statement.

**TABLE 1:** Core Competency domain gap areas as reported from site assessment.

#	Competency domain	% of all reported gaps
1	Scientific Concepts and Research Design	16.3
2	Ethical and Participant Safety Considerations	4.9
3	Investigational Products Development and Regulation	28.1
4	Clinical Study Operations	10.1
5	Study and Site Management	13.9
6	Data Management and Informatics	9.0
7	Leadership and Professionalism	8.0
8	Communication and Teamwork	9.7

The 3CTN PSC then evaluated which of the leveled competency statements from the summary report should be considered as priorities for Network-wide collaborative development strategies. Selection of an initial set of six leveled competency statements was based on prevalence of identified gaps, capacity for addressing each as well as potential impact for 3CTN’s strategic objectives for improving academic cancer trial conduct. Forty individual respondents from sites completed the follow-up survey and submitted scores based on relative need and importance. Respondents were typically research managers or research coordinators involved in the submission and management of the 3CTN grants along with their site’s leading investigator. Table 2 shows average scores, overall and by researcher role, associated with each leveled competency statement area. Ranking based on overall scores determined the final ranking of topics to be addressed first through Network-supported access to relevant learning opportunities, resource materials, educational workshops, or other initiatives.

**TABLE 2:** Network sites’ priority ranking of the six leveled competency statement gap areas selected by the PSC for initial Network-wide focus (n = 40 respondents).

Research role	# of respondents by role	Average score (1 = highest priority; 6 = not a priority)					
		Top priority leveled competency statements^a^					
		D4.1	D4.7	D4.8	D5.5	D6.1	D6.3
Clinical Research/Project Coordinator	7	2.00	2.43	2.29	1.86	2.43	1.86
Clinical Research Manager	18	2.89	2.72	2.83	2.17	3.67	2.17
Clinical Research Nurse	3	4.00	4.00	2.00	1.67	2.00	2.00
Investigator	5	4.40	3.60	3.20	3.40	3.60	2.40
Other^2^	7	3.71	3.00	2.71	2.43	3.57	2.14
**Average Score**		**3.15**	**2.93**	**2.70**	**2.28**	**3.30**	**2.13**
**Ranking**		**5**	**4**	**3**	**2**	**6**	**1**

^a^ Refer to detailed leveled competency statements in Supplementary Table S1, Supplementary Table S2 Research Associate, Research Program Manager, Quality Assurance Specialist, Research Database Coordinator, Canadian Cancer Trials Group (Collaborative trial group sponsor) representative, Network funder representatives (n = 2).

The bold values represents the Average Score (1 = highest priority; 6 = not a priority). Ranking: sequential ranking of priorities from 1 to 6, with 1 = highest priority and 6 = lowest priority.

## 4 Discussion

For 3CTN, maintaining and developing core competency levels across Canada’s academic cancer trial environment is required for realizing our strategic aims and progress on national-level initiatives. Therefore, along with working to address other factors that present challenges to sites’ sustained improvement in trial performance and capacity, supporting clinical research professional development was recognized as an essential activity. For sites in particular, an effective approach needs to be agile and foster ready access to resources relevant to diverse trial staff roles and responsibilities. Doing so better enables leadership to monitor and manage development within their trial teams. An approach based on the JTF CCF provided the level-setting standard for individual knowledge and skills assessment as well as a mechanism for gauging progress or other changes over time.

Clinical research sites typically rely on institutional training requirements mandated for clinical research professionals, accessing courses covering topics and guidelines such as Good Clinical Practice, Tri-Council Policy Statement 2 (TCPS 2), Introduction to Clinical Research, and Health Canada Food and Drug Regulations, Part C, Division 5 to supplement role orientation and on-the-job learning. Most Canadian sites have access to core training materials made available by their institutions or through memberships in established research networks in accordance with individual clinical research professional development plans. However, training course completion alone should not be taken to imply that the appropriate core competencies are in place to perform responsibilities fully compliant with applicable regulations and guidelines as well as highest ethical standards governing clinical research. Ascertaining core competency levels throughout an individual’s professional development is a crucial activity enabled by use of the JTF CCF as a standardized approach.

The 3CTN Core Competency Report facilitates this process by uniquely amalgamating available high-quality learning resources identified for each leveled competency statement into a single, functional tool. Accessible via our website, trial site managers can view report data at any time to enumerate and quantify workforce competency gaps. Professional development plans for clinical research professionals to address individual- or site-level gap areas can then be supported by directly accessing matched training resources of interest via links embedded within the report tool (Freel et al., 2023). The Report’s illustration of strengths and gaps for each domain filterable by site or province is designed to help inform resource development plans at the institutional, provincial, or national levels for 3CTN, its stakeholders and partners. For example, NCCs can use the 3CTN Core Competency Report to review and modify their onboarding plans and as part of their role to support trial unit operations at NACCs within their node, or as a basis for discussing joint training objectives.

Aggregate data obtained from all member sites at the beginning of our current 5-year strategy represented a snapshot of time and served as an initial step focused on identifying the general scale of competency gaps for each domain’s leveled competency statements. Initial site assessments did not therefore include a more in-depth reporting of competency level (i.e., Basic, Skilled or Advanced) related to each statement. In addition, the follow-up survey conducted to score priorities identified by the PSC did not include responses from members at all sites and the sample size was small for some clinical research roles. Recognizing these limitations, site clinical trial unit leaders subsequently participated in Network meetings to review and discuss findings as well as inform on tools needed to assist with training. A comprehensive evaluation is planned during the third year of our current strategy that will address cited limitations in order to provide a more complete picture for the Network that fully reflects all elements of the JTF framework. Results will also capture the number and proportion of sites that have incorporated use of the JTF framework and the 3CTN Core Competency Report in their staff development plans as well as provide insights on the effectiveness of Network initiatives implemented to support learning priorities identified by the PSC. The follow up site assessment may otherwise spotlight emerging areas that could benefit from new initiatives, training materials or other resources targeting unmet needs.

Connections with aligned organizations is essential for our success in this area, particularly where development of training content may be required. For example, Network contributions and member access to materials and mentoring opportunities arising from recent investments in Clinical Trials Training Platforms by the Canadian Institutes of Health Research as part of its Clinical Trials Fund (Canadian Institutes of Health Research, 2022).

For the remainder of its current strategic plan period, 3CTN aims to organize dedicated workshops with topics related to identified priority gap areas, either during annual stakeholder meetings or scheduled virtual webinars providing peer-to-peer learning platforms for member clinical research professionals from across Canada. As well, we will continue to collaborate on new initiatives and encourage ongoing assessment and reporting by sites to capture changes in workforce core competencies over time.

## Data Availability

The datasets presented in this study can be found in online repositories. The names of the repository/repositories and accession number(s) can be found in the article/Supplementary Material.
