# Supplementary material for: Minding the gaps: assessing and addressing clinical research core competencies across a network of Canadian cancer centres
Source: Front Pharmacol. 2023 Dec 8;14:1294335. doi: 10.3389/fphar.2023.1294335 (PMC10748401; doi:10.3389/fphar.2023.1294335)
Supplement: Supplementary file 1 [file DataSheet1.docx]

Supplementary Material

# Table S1. Results from the initial assessment from sites by Network Cancer Centres (NCC) and Network Affiliated Cancer Centres (NACC) (n=41). NACCs are linked to larger NCCs for resources to support their capacity to conduct academic trials.

| JTF Core Competency Domains & Leveled Competency Statements | Core Competency Gaps reported by Network Sites | | | |
| --- | --- | --- | --- | --- |
|  | NCC | | NACC | |
|  | #^1^ | %^2^ | #^1^ | %^2^ |
| Domain 1: Scientific Concepts and Research Design | | | | |
| D1.1 Apply principles of biomedical science to investigational product discovery and development and health-related behavioral interventions | 1 | 9.1% | 9 | 30% |
| D1.2 Identify scientific questions that are potentially testable clinical research hypotheses | 2 | 18.2% | 10 | 33% |
| D1.3 Identify the elements and explain the principles and processes of designing a clinical study | 0 | 0.0% | 7 | 23% |
| D1.4 Maintain awareness of new technologies, methodologies and techniques which enhance clinical study conduct, safety and validity | 0 | 0.0% | 0 | 0% |
| D1.5 Critically analyze clinical study results | 3 | 27.3% | 15 | 50% |
| Domain 2: Ethical and Participant Safety Considerations | | | | |
| D2.1 Differentiate between standard of care and clinical study activities | 1 | 9.1% | 0 | 0% |
| D2.2 Define the concepts of “clinical equipoise” and “therapeutic misconception” as they relate to clinical study conduct | 1 | 9.1% | 0 | 0% |
| D2.3 Apply relevant national and international principles of human participant protections and privacy throughout all stages of a clinical study | 0 | 0.0% | 0 | 0% |
| D2.4 Explain the evolution of the requirement for informed consent from research participants and the principles and content of key documents that help ensure the protection of human participants in clinical research | 0 | 0.0% | 1 | 3% |
| D2.5 Describe the ethical issues involved when dealing with vulnerable populations and what additional safeguards should be in place for those populations | 1 | 9.1% | 1 | 3% |
| D2.6 Evaluate and apply an understanding of the relevant ethical issues and cultural variation as it applies to the commercial aspects of the clinical research and investigational product development process | 3 | 27.3% | 4 | 13% |
| D2.7 Explain why inclusion, exclusion, and other criteria are included in a clinical protocol to assure participant protection | 0 | 0.0% | 1 | 3% |
| D2.8 Summarize the principles and methods of distributing and balancing risk and benefit; through selection and management of clinical study participants | 0 | 0.0% | 1 | 3% |
| Domain 3: Investigational Products Development and Regulation | | | | |
| D3.1 Discuss the historical events that precipitated the development of governmental regulatory processes for investigational products | 3 | 27.3% | 8 | 27% |
| D3.2 Describe the roles and responsibilities of the various institutions participating in the investigational products development process | 3 | 27.3% | 8 | 27% |
| D3.3 Explain the investigational products development process and the activities which integrate commercial realities into the life cycle management of medical products | 3 | 27.3% | 12 | 40% |
| D3.4 Summarize the legislative and regulatory framework that supports the development and registration of investigational products and ensures their safety, efficacy and quality | 2 | 18.2% | 7 | 23% |
| D3.5 Describe the specific processes and phases that must be followed for the regulatory authority to approve the marketing authorization for a medical product | 2 | 18.2% | 6 | 20% |
| D3.6 Describe the pre- and post- approval safety reporting requirements of regulatory agencies | 3 | 27.3% | 5 | 17% |
| D3.7 Appraise the issues generated and the effects of global expansion on the approval and regulation of medical products | 5 | 45.5% | 14 | 47% |
| Domain 4: Clinical Study Operations (GCPs) | | | | |
| D4.1 Explain how the design, purpose, and conduct of individual clinical studies fit into the goal of developing a new intervention | 1 | 9.1% | 2 | 7% |
| D4.2 Describe the roles and responsibilities of the clinical investigation team as defined by Good Clinical Practice Guideline | 0 | 0.0% | 0 | 0% |
| D4.3 Evaluate the design, conduct and documentation of clinical studies as required for compliance with Good Clinical Practice Guideline | 0 | 0.0% | 1 | 3% |
| D4.4 Compare and contrast the regulations and guidelines of global regulatory bodies relating to the conduct of clinical studies | 3 | 27.3% | 9 | 30% |
| D4.5 Describe appropriate control, storage and dispensing of investigational product | 0 | 0.0% | 0 | 0% |
| D4.6 Differentiate the types of adverse events (AEs) that may occur during clinical studies and explain the identification process and reporting requirement to IRBs/IECs, sponsors and regulatory authorities | 0 | 0.0% | 1 | 3% |
| D4.7 Describe how global regulations and guidelines assure human subject protection and privacy during the conduct of clinical studies | 1 | 9.1% | 5 | 17% |
| D4.8 Describe the role and process of monitoring a clinical study | 0 | 0.0% | 3 | 10% |
| D4.9 Describe the role and purpose of clinical study audits | 0 | 0.0% | 2 | 7% |
| D4.10 Describe the various methods by which safety issues are identified and managed in clinical studies | 0 | 0.0% | 1 | 3% |
| Domain 5: Study and Site Management | | | | |
| D5.1 Describe the methods used to determine whether to sponsor, supervise or participate in a clinical study | 0 | 0.0% | 4 | 13% |
| D5.2 Develop and manage the functional and operational efficiencies and personnel resources necessary to conduct a clinical study | 1 | 9.1% | 5 | 17% |
| D5.3 Describe the management and training approaches to mitigate risk to improve clinical study conduct | 0 | 0.0% | 6 | 20% |
| D5.4 Develop and implement strategies to manage participant recruitment, retention, compliance and track study activities | 1 | 9.1% | 6 | 20% |
| D5.5 Identify the legal responsibilities, liabilities and accountabilities that are involved in the conduct of clinical studies | 2 | 18.2% | 9 | 30% |
| D5.6 Identify and explain the specific procedural, documentation and oversight requirements of principal investigators, sponsors, CROs and regulatory authorities that relate to the conduct of a clinical study | 0 | 0.0% | 6 | 20% |
| D5.7 Identify, organize, analyze and report project performance for comprehensive management of a clinical study | 0 | 0.0% | 0 | 0% |
| Domain 6: Data Management and Informatics | | | | |
| D6.1 Describe the role and importance of statistics and informatics in clinical studies | 3 | 27.3% | 3 | 10% |
| D6.2 Describe the origin, flow, and management of data through a clinical study | 0 | 0.0% | 0 | 0% |
| D6.3 Describe best practices and resources required for standardizing data collection, capture, management, analysis, and reporting | 3 | 27.3% | 7 | 23% |
| D6.4 Describe, develop, and implement processes for data quality assurance | 3 | 27.3% | 7 | 23% |
| Domain 7: Leadership and Professionalism | | | | |
| D7.1 Describe and apply the principles and practices of leadership, management and mentorship in clinical research. | 2 | 18.2% | 5 | 17% |
| D7.2 Identify ethical and professional conflicts associated with the conduct of clinical studies and implement procedures for their prevention or management | 2 | 18.2% | 2 | 7% |
| D7.3 Identify and apply the professional guidelines and codes of ethics that apply to the conduct of clinical research | 0 | 0.0% | 0 | 0% |
| D7.4 Describe the impact of regional diversity and demonstrate cultural competency in clinical study design and conduct | 3 | 27.3% | 9 | 30% |
| Domain 8: Communication and Teamwork | | | | |
| D8.1 Describe the importance of team science and methods necessary to work effectively with cross-functional, multidisciplinary and inter-professional research teams, which may include external partners | 1 | 9.1% | 2 | 7% |
| D8.2 Discuss the relationship and appropriate communication between Sponsor, CRO and clinical research site | 2 | 18.2% | 0 | 0% |
| D8.3 Effectively communicate the content and relevance of clinical research findings to colleagues, advocacy groups and the non-scientist community | 5 | 45.5% | 9 | 30% |
| D8.4 Describe the components of a traditional scientific publication | 1 | 9.1% | 8 | 27% |

^1^ Number of respondents who indicated a gap, defined as being incapable of performing activities described for each leveled competency statement.

^2^ Proportion of the total number of respondents that identified the level competency statement as a gap area.

**Table S2. Core competency self-assessment survey completed by cancer centres at time of 3CTN membership application.**

| **Core Competency Domain** | **Diagnostic Question** | **Competency Statements** |
| --- | --- | --- |
| **Example Domain** | **Example Question** | **Example Statement** |
| **Scientific Concepts and Research Design**  Types of Tasks: • Preparation for research study • In-house study development | Do your clinical trial staff members possess a knowledge of scientific concepts related to the design and analysis of clinical trials? | Apply principles of biomedical science to investigational product discovery and development and health-related behavioral interventions |
|  |  | Identify scientific questions that are potentially testable clinical research hypotheses |
|  |  | Identify the elements and explain the principles and processes of designing a clinical study |
|  |  | Critically analyze clinical study results |
| **Ethical and Participant Safety Considerations**  Types of Tasks: • REB submissions | Do your clinical trial staff members place importance on the care of patients, aspects of human subject protection, and safety in the conduct of a clinical trial? | Differentiate between standard of care and clinical study activities |
|  |  | Define the concepts of “clinical equipoise” and “therapeutic misconception” as they relate to the conduct of a clinical study |
|  |  | Apply relevant national and international principles of human subject protections and privacy throughout all stages of a clinical study |
|  |  | Explain the evolution of the requirement for informed consent from research participants and the principles and content of the key documents that ensure the protection of human participants in clinical research |
|  |  | Describe the ethical issues involved when dealing with vulnerable populations and what additional safeguards should be in place for those populations |
|  |  | Evaluate and apply an understanding of the relevant ethical issues and cultural variation as it applies to the commercial aspects of the clinical research and investigational product development process |
|  |  | Explain why inclusion, exclusion, and other criteria are included in a clinical protocol to assure human subject protection |
|  |  | Summarize the principles and methods of distributing and balancing risk and benefit; through selection and management of clinical study subjects |
| **Investigational Products Development and Regulation**  Types of Tasks: • SOPs and compliance • Specimen handling, biobanking | Do your clinical trial staff members understand how investigational products are developed and regulated? | Discuss the historical events that precipitated the development of governmental regulatory processes for investigational products |
|  |  | Describe the roles and responsibilities of the various institutions participating in the investigational products development process |
|  |  | Explain the investigational products development process and the activities which integrate commercial realities into the life cycle management of medical products |
|  |  | Summarize the legislative and regulatory framework that supports the development and registration of investigational products and ensures their safety, efficacy and quality |
|  |  | Describe the specific processes and phases that must be followed for the regulatory authority to approve the marketing authorization for a medical product |
|  |  | Describe the pre- and post- approval safety reporting requirements of regulatory agencies |
|  |  | Appraise the issues generated and the effects of global expansion on the approval and regulation of medical products |
| **Clinical Trial Operations (GCP)**  Types of Tasks: • SOPs and training of staff, compliance | Do your clinical trial staff members possess an understanding of study management and GCP compliance; safety management (adverse event identification and reporting, post-market surveillance, and pharmacovigilance), and handling of investigational product? | Explain how the design, purpose, and conduct of individual clinical studies fit into the goal of developing a new intervention |
|  |  | Describe the roles and responsibilities of the clinical investigation team as defined by Good Clinical Practice Guidelines |
|  |  | Evaluate the design, conduct and documentation of clinical studies as required for compliance with Good Clinical Practice Guidelines |
|  |  | Compare and contrast the regulations and guidelines of global regulatory bodies relating to the conduct of clinical studies |
|  |  | Describe appropriate control, storage and dispensing of investigational product |
|  |  | Differentiate the types of adverse events (AEs) that may occur during clinical studies and explain the identification process and reporting requirement to IRBs/IECs, sponsors and regulatory authorities |
|  |  | Describe how global regulations and guidelines assure human subject protection and privacy during the conduct of clinical studies |
|  |  | Describe the role and process of monitoring a clinical study |
|  |  | Describe the role and purpose of clinical study audits |
|  |  | Describe the various methods by which safety issues are identified and managed in clinical studies |
| **Study and Site Management**  Types of Tasks: • Metrics for study performance • Tasks specific to developing  studies in-house (IIT) • Oversight of  study conduct | Do your clinical trial staff members understand content required at the site level to run a study (financial and personnel aspects), Including site and study operations (not encompassing regulatory/GCPs)? | Describe the methods used to determine whether to sponsor, supervise or participate in a clinical study |
|  |  | Develop and manage the financial, timeline, and personnel resources necessary to conduct a clinical study |
|  |  | Describe the management and training approaches to mitigate risk to improve clinical study conduct |
|  |  | Develop strategies to manage participant recruitment, retention, compliance and track study activities. |
|  |  | Identify the legal responsibilities, liabilities and accountabilities that are involved in the conduct of clinical studies |
|  |  | Identify and explain the specific procedural, documentation and oversight requirements of principal investigators, sponsors, CROs and regulatory authorities that relate to the conduct of a clinical study |
| **Data Management and Informatics**  Types of Tasks: • Data collection and retention procedures • Data quality  • CTMS related activities • Training on data and privacy related issues | Do your clinical trial staff members understand how data are acquired and managed during a clinical trial, including source data, data entry, queries, quality control, and correction and the concept of a locked database? | Describe the role and importance of statistics and informatics in clinical studies |
|  |  | Describe the origin, flow, and management of data through a clinical study |
|  |  | Describe best practices and resources required for standardizing data collection, capture, management, analysis, and reporting |
|  |  | Describe, develop, and implement processes for data quality assurance |
| **Leadership and Professionalism**  Types of Tasks: • Professional development of staff • Collaboration with other research sites | Do your clinical trial staff members understand the principles and practice of leadership and professionalism in clinical research? | Describe and apply the principles and practices of leadership, management and mentorship in clinical research. |
|  |  | Identify ethical and professional conflicts associated with the conduct of clinical studies and implement procedures for their prevention or management. |
|  |  | Identify and apply the professional guidelines and codes of ethics that apply to the conduct of clinical research. |
|  |  | Describe the impact of regional diversity and demonstrate cultural competency in clinical study design and conduct |
| **Communication and Teamwork**  Types of Tasks: • Team building activities • Engaging site staff, and physician engagement • Flow of communication at the site | Are your clinical trial staff members able to demonstrate all elements of communication within the site, between the site and sponsor, CRO, and regulators? | Discuss the relationship and appropriate communication between Sponsor, CRO and clinical research site. |
|  |  | Describe the components of a traditional scientific publication. |
|  |  | Effectively communicate the content and relevance of clinical research findings to colleagues, advocacy groups and the non-scientist community. |
|  |  | Describe the importance of team science and methods necessary to work effectively with multidisciplinary and inter-professional research teams. |

Table S3. Follow up survey to score priorities for each of the competency statements identified by the Performance Strategy Sub-Committee.

| **#** | **Question** |
| --- | --- |
|  | **What is your current role?**  (Investigator, Clinical Research Manager, Clinical Research/Project Coordinator, Clinical Research Nurse, Patient Representative, Other) |
|  | **Which province are you located in?**  (British Columbia, Alberta, Saskatchewan, Manitoba, Ontario, Quebec, Nova Scotia, Newfoundland) |
|  | **Cancer Centre patient population:**  (Adults, Pediatrics) |
|  | **Core Competency Framework for Clinical Research Professionals**  Implementation of the [Joint Task Force Core Competency Framework](https://mrctcenter.org/clinical-trial-competency/) aims to promote the capacity for, high-quality cancer clinical research across the Network. Trial staff identified gaps in core competencies, as part of the 3CTN 2022 Request for Applications process and the results were presented to the Performance Strategy Sub-Committee (PSC) in June. Next steps involve conducting an environmental scan for available resources and to make training programs in priority areas available across the Network.  The responses to the following questions will be used to further prioritize core competencies areas for 3CTN to address. |
|  | Based on the identified Network core competency gaps, the 3CTN Performance Strategy Sub-Committee identified the following core competency statements as *priorities to address* across the Network.  Indicate the priority of each statement based on its significance to your role/team’s performance and would benefit from additional training:  (Highest priority = 1, High priority = 2, Medium priority = 3, Low priority = 4, Lowest priority = 5, Not a priority = 6)   1. **Clinical Study Operations (Good Clinical Practice):** Explain how the design, purpose, and conduct of individual clinical studies fit into the goal of developing a new intervention 2. **Clinical Study Operations (Good Clinical Practice):** Describe how global regulations and guidelines assure human subject protection and privacy during the conduct of clinical studies 3. **Clinical Study Operations (Good Clinical Practice):** Describe the role and process of monitoring a clinical study 4. **Study and Site Management:** Identify the legal responsibilities, liabilities and accountabilities that are involved in the conduct of clinical studies 5. **Data Management and Informatics:** Describe the role and importance of statistics and informatics in clinical studies 6. **Data Management and Informatics:** Describe best practices and resources required for standardizing data collection, capture, management, analysis, and reporting |
|  | Suggestions for additional training topics: |
